# Care needs of patients with the post-COVID syndrome in Dutch general practice: an interview study among patients and general practitioners

**DOI:** 10.1186/s12875-024-02597-w

**Published:** 2024-09-28

**Authors:** Corinne Rijpkema, Bart J. Knottnerus, Rinske van den Hoek, Lisa Bosman, Liset van Dijk, Robert A. Verheij, Isabelle Bos

**Affiliations:** 1 Nivel, Netherlands Institute for Health Services Research, Otterstraat 118, Utrecht, 3513CR The Netherlands; 2 Tranzo, Tilburg School of Social and Behavioural Sciences, Tilburg University, Tilburg, The Netherlands; 3 Department of Surgery, University of Groningen, University Medical Center Groningen, Groningen, The Netherlands; 4 Department of PharmacoTherapy, -Epidemiology & -Economics (PTEE), Groningen Research Institute of Pharmacy, Faculty of Science and Engineering, University of Groningen, Groningen, The Netherlands

**Keywords:** Post-COVID syndrome, COVID-19, General practice, Qualitative research, Appropriate care, Care continuity, Patient experiences

## Abstract

**Background:**

The post-COVID syndrome (PCS) has a large impact on an individual’s daily life. The wide variety of symptoms in PCS patients and the fact that it is still relatively new makes it difficult for general practitioners (GPs) to recognize, diagnose and treat patients with PCS, leading to difficulties in assessing and fulfilling healthcare needs. It is largely unknown what the experiences of Dutch patients and GPs are with PCS and, therefore, we gained insight into the different aspects of living with PCS and the associated healthcare needs.

**Methods:**

Semi-structured interviews were performed with 13 self-reported PCS patients (varying in sex, age, education, and health literacy) and 6 GPs (varying in gender, age, and type of practice) between January-July 2022. Patients and GPs were most likely unrelated (not in the same practices). The data have been analysed using the Thematic Analysis method.

**Results:**

Experiences appeared to vary between two types of PCS patients that emerged during the interviews: (1) individuals with *good pre-existing health status (PEHS)* who are severely affected by PCS and have difficulty recovering and (2) individuals with *poorer PEHS* whose health became even poorer after COVID-19 infection. The interviews with PCS patients and GPs revealed two main themes, in which the types of patients differed: (1) *aspects of living with PCS*; individuals with good PEHS mainly experience symptoms when overstimulated, while individuals with poorer PEHS generally feel exhausted continuously. (2) *Healthcare experiences*; GPs emphasized that individuals with good PEHS seem to benefit from support in distributing their energy by careful planning of daily activities, whereas individuals with poorer PEHS require support in activation. Patients and GPs emphasised the importance of taking patients seriously and acknowledging their symptoms. Finally, the patients interviewed indicated that some GPs doubted the existence of PCS, resulting in insufficient recognition.

**Conclusion:**

Awareness of the differences in needs and experiences of the two types of PCS patients could contribute to more appropriate care. Acknowledgement of PCS by GPs as a real syndrome is important for patients and plays an important role in coping with or recovering from PCS. A multidisciplinary person-centred approach is important and can be coordinated by a GP.

**Supplementary Information:**

The online version contains supplementary material available at 10.1186/s12875-024-02597-w.

## Background

The COVID-19 pandemic has had a significant impact on public health and society. This is partly due to the measures implemented to combat SARS-CoV-2, such as social distancing and lockdowns [1], but also through the consequences for individuals infected with SARS-CoV-2 [2]. Up until April 2023, more than 700 million people worldwide and more than 8 million people in the Netherlands have been reported as infected with SARS-CoV-2, known as COVID-19 [3]. There is a wide variety of symptoms reported to be associated with COVID-19, as some people undergo an asymptomatic infection, while others experience symptoms ranging from respiratory symptoms to multi-organ failure [4, 5]. A substantial proportion of symptomatic patients (reported range of 4.7–80%) have persistent symptoms, also called post-COVID syndrome (PCS) [6, 7]. Over one-third of these PCS patients have various pre-existing conditions [8]. Patients with PCS suffer from a variety of symptoms; e.g. chronic fatigue, shortness of breath, sleep disorders, chest pain, difficulties with concentration, memory disturbance, or an altered sense of smell [9–12]. These symptoms often have a serious impact on patient’s daily life, as basic daily activities have drastically declined for them [13]. In addition, patients fear about the course of their symptoms, about recovery and stigmatization by healthcare providers, employers and others in the community [14, 15].

Due to the wide variety of symptoms and the fact that PCS is still a fairly new phenomenon, it is sometimes difficult for general practitioners (GPs) to recognize, diagnose and treat patients with PCS, leading to difficulties in assessing and fulfilling their healthcare needs. Patients with PCS experienced difficulty accessing care as GPs indicated their inability to help them, resulting in frustration and a sense that GPs were not caring [14]. Moreover, the variation in symptoms and pre-existing conditions result in a wide variety of care needs for patients and no suitable care pathways have been defined for PCS [13, 14]. Nonetheless, there is scepticism among healthcare professionals regarding the existence of PCS [16]. This results in a lack of recognition for patients, leading to implications for delayed diagnosis and deferred treatment [16]. In the Netherlands, GPs are the first point of contact for patients; they assess the physical and mental symptoms, and together with patients, GPs and patients jointly decide what care would be needed taking into account one’s medical history [17]. Moreover, GPs play a gatekeeping role regarding access to specialized secondary care [17] and thus play an important role in assessing PCS patients’ healthcare needs. For further development of appropriate care for this group of patients, it is important to learn about their care experiences thus far.

However, there are only a few qualitative studies on the experiences of patients and GPs, and the perspective is still unclear in the Netherlands. Therefore, this study aimed to gain insight into the experiences of Dutch PCS patients and GPs with regard to living with PCS as well as their experiences with PCS care. These experiences can be used to provide practical guidance on providing care to patients with PCS.

## Methods

### Study setting and participants

We performed a descriptive qualitative study based on a phenomenological approach [18] which enabled us to explore the experiences of post-COVID syndrome (PCS) patients and GPs regarding living with PCS and the care for these patients. For this study, we purposefully selected patients and GPs, and subsequently recruited two additional patients through snowball sampling [19, 20]. Recruitment stopped after data saturation was experienced by the interviewing researcher.

### Patient selection

Patients were recruited from the Nivel Corona Cohort, a longitudinal cohort study of COVID-19 patients initiated in 2020 [21]. Patients recruited for this cohort had been asked about their willingness to participate in further (qualitative) research. A selection of these patients was invited to participate in the current study. Patients who self-reported persistent symptoms three months or more after their reported COVID-19 infection were selected from the cohort and defined as having PCS, as used by the World Health Organisation [22]. They were selected for variety in gender, age, education, duration of symptoms, and health literacy (based on Chew’s Set of Brief Screening Questions – SBSQ) [23]. Patients were excluded if they did not report any symptoms present at the time of recruitment. Selected patients were approached by e-mail by the interviewing researcher (BK). Consenting patients could choose between an online or a telephone interview. Two participants declined because they were unable to participate in the interview as a result of their PCS symptoms.

### General practitioners selection

General practitioners were recruited from the general practices that take part in Nivel Primary Care Database (Nivel-PCD) [24], a database holding extracts from general practices’ electronic health records. A selection of 8 GPs were invited to participate in the study. The selection criterion was variation in practice type (solo, duo, or group). Additional GPs were sought via a periodic newsletter among all Nivel-PCD. When GPs indicated their willingness to participate in an interview, the interviewing researcher (BK) contacted the GP by e-mail to schedule an appointment for the interview. There were no exclusion criteria.

Patients and GPs were most likely unrelated (not in the same practices), due to being recruited in different ways and considering the large population from which they were recruited. For both patients and GPs, additional information about the study and the interview procedure was provided before the interview by e-mail. Written (digitally) informed consent was obtained from each participant prior to the interview. All interviewees received a 50 euro gift card. After the interviews, the interviewing researcher removed all e-mail addresses and correspondences with participants.

### Data collection

One experienced interviewer and GP (BK), conducted a total of 19 interviews between January and July 2022: six with GPs and 13 with patients. All six interviews with GPs were held online. For patients, nine interviews were held online and four by telephone. Due to restrictions related to the COVID-19 pandemic, interviews could not be conducted in person. Only the participant and interviewer were present during the interviews. There were no professional or personal relationships between the participants and the interviewer.

Semi-structured interviews were conducted using a topic list, which consisted of open-ended questions. Two topic lists were developed, one for patients and one for GPs, (see Additional file 1). The topic list for patients was based on the guideline from The Dutch College of General Practitioners (NHG) for medically unexplained physical symptoms (MUPS), in Dutch SOLK [25]. This guideline involves five different dimensions of symptoms that need to be addressed: somatic, cognitive, emotional, behavioural, and social [25], which is in line with the Dutch PCS guideline [26]. These dimensions were included in the topic list, together with additional questions about patients’ experience with acute COVID and their experiences regarding professional and informal care.

The topic list for GPs was based on the research question about the experiences with care for (vulnerable) PSC patients and the barriers they have encountered. The topic list consisted of five main topics: (1) the experiences with care for PCS patients, (2) communication with PCS patients, (3) risk groups for PCS, (4) collaboration with other healthcare disciplines and (5) barriers and facilitators.

Both topic lists were constructed by members of the research team. Pilot versions of the topic lists were reviewed by two experts in qualitative research, who were diagnosed with PCS themselves. At the end of the interviews, participants were asked if they had any additional topics for discussion.

Interviews with patients lasted approximately 60 min and with GPs approximately 30 min. The interviews were all audio-recorded and (field)notes were made after each interview that highlighted the key points discussed. During the coding process, it was ensured that these points were included in the codes. No summary of the interview was sent to the participants. However, during the interviews, the information mentioned by patients and GPs was regularly summarized and clarified by the interviewer for correct interpretation.

### Data analysis

The audio recordings were transcribed verbatim. The transcripts were coded by one researcher (BK). With three other researchers (CR, LB, RvdH), two transcripts of patient interviews and two transcripts of GPs were discussed until a consensus was reached on the codes. After coding all interviews, another researcher (CR) checked the codes for twelve interviews, both for the patient and GP interviews. The method of Braun and Clarke [27] was used for the Thematic Analysis of the data, in which themes were derived from the data [28]. According to this method, researchers started with the open coding process, generating initial codes and categories that describe characteristics of potentially relevant data. After that, the axial coding process started, in which codes were divided into overarching (sub)themes, based on the framework of MacPherson, et al. [15]. Finally, selective coding was performed; codes were modified and ordered, duplicates were removed and the overarching themes were integrated into theories. The steps in the data analysis process were iterative. As described above, multiple researchers were involved in the data analysis process, leading us to adopt a reflexive approach [29]. This was done to minimize assumptions by reflecting on what we identified and inferred, and then examining whether those assumptions applied to specific themes [29]. This approach ensured a comprehensive and more nuanced interpretation of the data [29]. The data were analysed using MAXQDA version 2022 [30]. This manuscript was reported according to the Consolidated Criteria for Reporting Qualitative research (COREQ) checklist for reporting qualitative research [31].

## Results

**Fig. 1:**
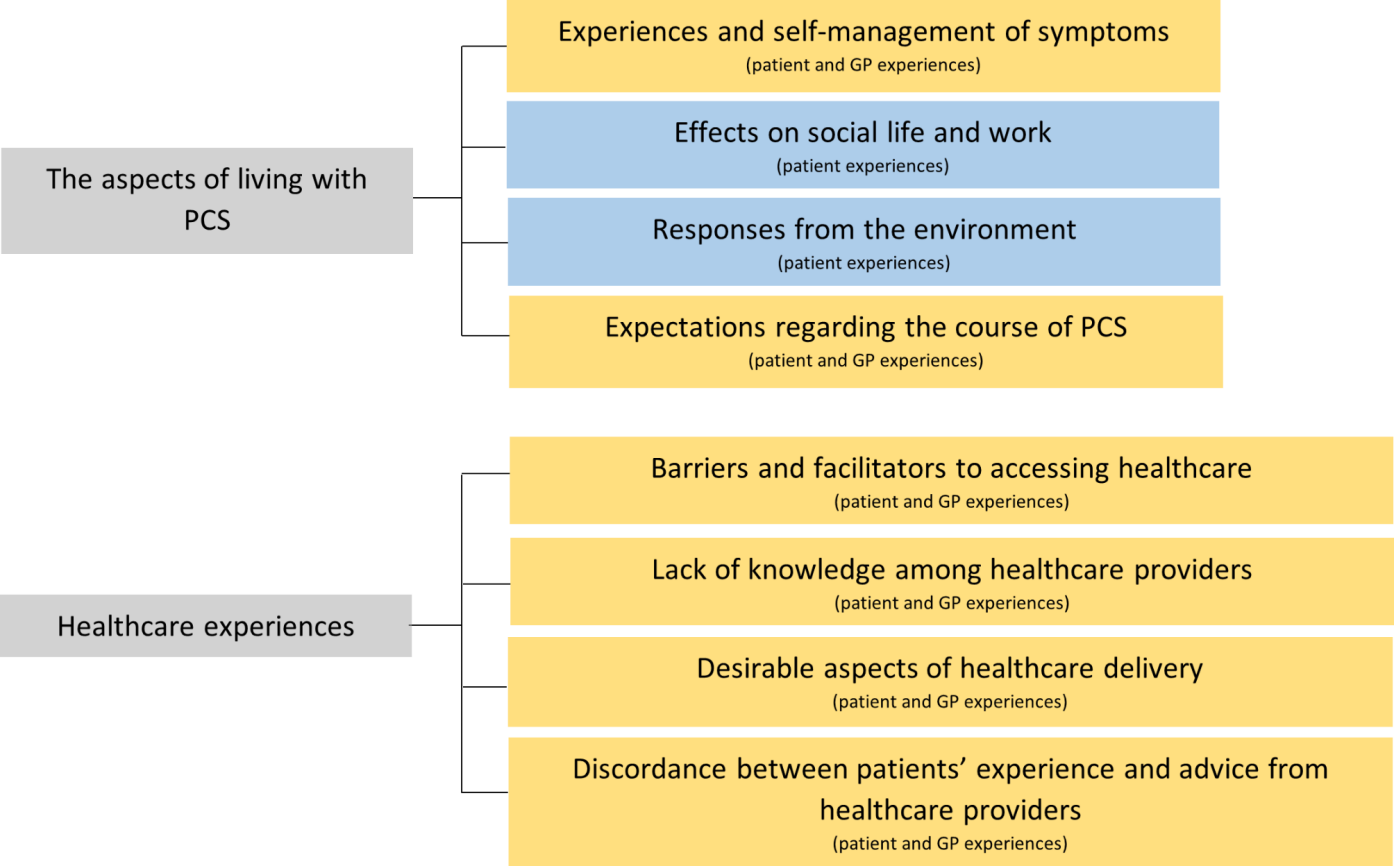
Themes and subthemes (yellow: experiences of patients and GPs, blue: experiences of patients)

In total, 19 interviews were conducted: 13 with post-COVID syndrome (PCS) patients (Table 1), and 6 with GPs (Table 2). All patients were in paid employment before developing PCS and none of the patients had a migration background. Two patients had completed secondary education, three had completed secondary vocational education, and eight had completed university of applied sciences or university education. Additionally, two patients had symptoms for less than one year, eight had symptoms for 1 to 2 years, and three had symptoms for more than two years. Nine patients had adequate health literacy, while four had limited health literacy. During the iterative process of analysing the interviews, two groups of patients emerged who have in some cases different experiences/needs with PCS: (1) individuals that self-reported good pre-existing health status (PEHS) and (2) individuals that self-reported poorer PEHS (see Table 1). By ‘health’ we mean physically and mentally healthy and no frailty. The pre-defined (sub-)themes; (1) the aspects of living with PCS (e.g. self-management, responses from the environment and effects on social life and work) and (2) healthcare experiences (e.g. barriers/facilitators to accessing healthcare), are described below and displayed in Fig. 1.

**Table 1:** Patient characteristics

ID	Age	Gender	Urbanity residence	Civil status	Health status before PCS
1	33	Male	Village	Single	Healthy
2	51	Female	Village	Married - two children	Endometriosis and fatigue
3	24	Female	Village	Single	Migraine
4	63	Male	City	Married	Chronic lymphocytic leukaemia
5	41	Male	City	Married - two children	Crohn’s disease
6	54	Female	City	Partner - two children	Healthy
7	52	Male	Village	Married - two children	Healthy
8	53	Female	Village	Married - three children	High blood pressure
9	49	Female	Village	Partner	Exercise-induced asthma
10	65	Female	Village	Married - four children	Intestinal symptoms
11	45	Female	Village	Married - two children	Healthy
12	48	Male	City	Partner - one child	Allergies, postural orthostatic tachycardia syndrome, collagen problems, asthma
13	35	Female	City	Married - one child	Healthy

**Table 2:** General practitioner’s characteristics

ID	Age	Gender	Work experiences (years)	Urbanity	Type of practice	Patient population according to GP in socioeconomic status (SES)
1	46	Female	17	Village	Duo	Mostly high SES
2	49	Male	13	Big village	Single-handed	Average SES
3	65	Male	36	City	Group	Low SES
4	42	Female	6	City	Group	Average SES
5	49	Male	20	Big village	Group	Average SES
6	60	Male	27	City	Group	Average SES

### The aspects of living with PCS

#### Experiences and self-management of symptoms

##### Patients

Most patients with poorer PEHS mentioned their pre-existing conditions as a possible explanation for their persistent symptoms. They regarded some of these conditions, like chronic lymphocytic leukaemia, Crohn’s disease, or asthma, to have had a negative impact on the development of their PCS symptoms. Other patients had a good PEHS and had no explanation for their persisting symptoms.

*“I would not know. Is it my age? Is it because I no longer have a uterus? Is it because of menopause? Is it hormonal? I have no idea why I have had symptoms for so long.”* – patient with good PEHS [6].

Both PEHS groups reported diverse PCS symptoms after a COVID-19 infection, from extreme fatigue to concentration problems and, therefore, experienced their symptoms very differently.

*“I do not experience the fatigue that other PCS patients sometimes describe. If there has been a lot going on in my life that I need to process*,* then I suffer from fatigue more quickly.”* – patient with good PEHS [13].

*“Even small things take a lot of energy. So I got up early in the morning and made breakfast for my children. I had breakfast myself*,* I just read the newspaper. Usually*,* I was already tired by then and I went back to bed. It was always: you go to bed tired*,* but you also wake up tired.” –* patient with poorer PEHS [5].

Some patients with poorer PEHS found the persistent symptoms elusive, resulting in anxiety about the cause of the disease. For both groups, some patients mentioned that a second infection with SARS-CoV-2 caused a relapse of (new) symptoms. According to patients remembering things was sometimes difficult.

*“Occasionally*,* when someone poses a question*,* I find myself thinking: “What was the question again?”. Similarly*,* when my thoughts become jumbled and overwhelming*,* I may need a moment to catch up and regain my focus.”*– patient with poorer PEHS [8].

Patients mentioned different ways of self-managing their symptoms. Both groups indicated that some behaviour reduces PCS symptoms. For example, a daily or weekly activity schedule helped to spread activities throughout the day and alternated with sleeping and resting, which reduced symptoms such as fatigue.

*“At one point*,* I especially noticed that every afternoon after lunch*,* I would lie down on the couch for an hour*,* and then I would sleep for a while. Then*,* I would get through the afternoon without feeling very tired.”* – patient with good PEHS [7].

Moreover, PCS has helped certain overweight patients recognize the significance of healthy living, such as consuming fewer calories and engaging in more physical activity, resulting in weight loss. However, some patients with poorer PEHS did everything to improve stamina (e.g. on a home trainer, treadmill, or elastic exercises), but their behaviour did not reduce PCS symptoms, leading to a feeling of no control. Nevertheless, there were also exceptions, for example, a patient with poorer PEHS noted that PCS symptoms got worse when limits were exceeded during physical activity, such as high-intensity interval training, while a patient with good PEHS already experienced an increase in PCS symptoms when climbing stairs.

##### General practitioners

The GPs agreed with patients that most PCS patients already suffered from pre-existent, sometimes unexplained, health problems and, therefore, recognized the relationship with PEHS. Nevertheless, some GPs emphasized that a minority of the patients had no pre-existing health problems and that it is unpredictable who gets PCS and who does not. In addition, GPs described the group with a good PEHS as individuals who are healthy and young, severely affected by PCS and have difficulty recovering. The group with poorer PEHS was described by GPs as individuals who were already low on energy before COVID-19 and became even more tired after.

*“It concerns someone who is young*,* who has always been active in sports … It’s the kind of situation where you just do not expect someone not to recover well … In addition to the people who already felt life was difficult*,* had no energy for anything. Getting COVID-19 on top of that and being even more exhausted than they already were.”* – general practitioner [4].

One GP, therefore, suggested that the underlying pathology might also differ between these two groups.

According to some GPs, most patients were able to self-manage PCS. They noted that patients’ knowledge of PCS increased over time, which made patients more self-reliant and could, therefore, better access the necessary care. However, some GPs mentioned that patients misused the PCS label to get free access via a GP referral to another healthcare provider, for example, to receive physiotherapy.

#### Effects on social life and work

##### Patients

Patients’ social lives were also affected by PCS. Due to PCS, patients from both groups shortened their visits to family and friends because it takes too much energy or they wanted to avoid crowds. Some patients mentioned that going on a holiday would take a lot of energy. Most patients from both groups accepted that their social lives had changed and tried to live with their physical and mental limitations, even though it made them feel worthless, angry, and frustrated. Some other patients from both groups emphasized that after a long time, they had resumed their social lives, as they could exercise or enjoy a night out again. Some patients from both groups emphasized the importance of staying socially active and that it helped to talk to friends and family about what PCS was doing to them.

*“It has helped to just talk to people who know you well*,* colleagues*,* best friends or family about what PCS is doing to you. That helps to keep life with PCS bearable.”* – patient with good PEHS [13].

Patients’ work was significantly impacted by PCS. Most patients from both groups indicated that they could not work all day because of concentration problems due to PCS. This led to stress and uncertainties for patients. One patient indicated that her employer provided little cooperativeness to return to work.

*“I have been indicating for three months now that I want to do something and that I want to discover what I can do at work*,* instead of always being told what I cannot do. […] it is difficult if you do not receive the support to do so.”* – patient with poorer PEHS [9].

A patient with good PEHS feared whether she would ever be able to return to work full-time. Some patients from both groups mentioned that they were no longer able to work at all; could no longer practice their profession or take early retirement. Nevertheless, some patients from both groups were able to work full-time again after PCS; a patient with good PEHS now works on fixed days and times to conserve energy.

#### Responses from the environment

##### Patients

For both groups, most patients received and appreciated support and understanding from their relatives. This created a positive feeling about recovery. Most PCS patients from both groups mentioned that they were members of a support group on social media (such as Facebook), have joined an organization that supports people with long-term COVID symptoms (C-support) [32], or had contact with other patients during physical therapy or rehabilitation. Recognition and mutual understanding were helpful for patients.

*“Sometimes it is good to talk to each other about experiences*,* for example how the other person experiences PCS symptoms*,* then you feel each other well. And you can help others with that too.”* – patient with good PEHS [11].

Most patients from both groups felt that people who were not close to them did not understand their illness. Some people belittled the PCS symptoms by saying that they also experienced fatigue and that it could be remedied by getting a good night’s sleep. Patients with PCS often received unsolicited advice and experienced that others did not listen carefully to their feelings.

*“People say: “It would be really good for you if you could take a walk outside every day before breakfast.” And then I try to explain that it already takes a lot of energy and effort to get out of bed at all*,* to make my sandwich and get dressed. That I really cannot walk outside for a while.”* – patient with poorer PEHS [9].

Some patients with poorer PEHS preferred not to talk about their PCS symptoms to avoid misunderstandings. A patient with poorer PEHS indicated that it was important that the diagnostic process should be improved for PCS because once there is a well-defined label, there will be more understanding at a societal level.

#### Expectations regarding the course of PCS

##### Patients

Most patients from both groups expected to maintain mild residual PCS symptoms but were happy if they could function normally again in daily life, even if some symptoms remained. However, some patients with a good PEHS found it difficult that they are not physically at the level they were performing before the infection and found it difficult to accept that there were persistent symptoms. This made patients uncertain about their future lives.

*“I have done everything I could do. I do not know if I will fully recover. I have actually kind of given up hope already. I am still making progress*,* but it is excruciatingly slow. Six months ago I said: “If I become seventy percent of myself*,* then I am happy.” But I am just not fully recovered and that is frustrating.”* – patient with good PEHS [13].

##### General practitioners

According to the GPs, they also mentioned that when patients regained function (i.e. were able to go to work or have enough energy to meet up with friends), and could accept the presence of the symptoms, they considered someone recovered.

*“When the patient says: “The only thing I notice is that I am a bit short of breath when exerting myself.” The effort level of this patient seems pretty good to me*,* if she can walk 10 km every day*,* then she can do something. So yes*, *then the patient has recovered reasonably well.”* – general practitioner [2].

A GP mentioned that there were people who recovered from PCS without any problems within a short period, but most patients recovered in a longer period, for example, after a year or sometimes even longer. This GP compared PCS to post-viral fatigue syndrome after Epstein Barr or Lyme infection. In communication with patients, some GPs indicated that symptoms can persist for a long time after the infection, but that they can recover well from it. However, another GP did not emphasize this in communication to patients because there is no benefit to patients knowing this. Moreover, GPs emphasized the significance of providing patients with a sense of perspective by assuring them that the majority of PCS patients recover and that progress comes in small steps, as a result, patients accepted their symptoms better.

### Healthcare experiences

In general, PCS patients consulted many different healthcare providers; the general practitioner, nurse practitioner (a nurse who works in a GP office), physiotherapist, occupational therapist, pulmonologist, cardiologist, rehabilitation physician, and dietician. This resulted in different experiences with healthcare.

#### Barriers and facilitators to accessing healthcare

##### Patients

Both patient groups identified various barriers and facilitators related to accessing healthcare services. They mentioned that their symptoms sometimes hindered their ability to receive care or follow medical advice. For example, patients reported feeling fatigued after travelling to see a physiotherapist or experiencing difficulty following exercise recommendations due to fatigue.

*“At one point*,* I also went to a physiotherapist. And yes*,* I did that*,* but it was very difficult to schedule consistently*,* because maybe you wanted to go for a walk*,* but then you were so tired that it just was not possible.” – patient with poorer PEHS [5]*.

Additionally, patients expressed difficulties in reaching their GPs, encountered long waiting times for specialized care as a result of acute COVID-19 care, and noted that the reimbursement for physiotherapy care was inadequate. Other patients from both groups highlighted that they had no trouble accessing physiotherapists or occupational therapists. Patients with higher levels of education felt more capable of navigating the healthcare system and obtaining appropriate support.

##### General practitioners

Similar to PCS patients, GPs also indicated some barriers for PCS patients to receive care, for instance, long waiting times made it challenging for GPs to refer patients to other healthcare professionals.

*“If they need long-term psychological help as they are stuck in a work situation*,* family situation*, etc.,* the waiting times for mental healthcare are quite dramatic at the moment.” –* general practitioner [5].

Most GPs indicated that they had the feeling that they did not have a complete image of which of their patients had PCS because not all patients consulted them. According to the GPs, this might be due to patients feeling hesitant to approach them, as some patients mentioned to the GPs that they felt that GPs were busy during the COVID-19 pandemic.

#### Lack of knowledge among healthcare providers

##### Patients

Most patients with poorer PEHS mentioned that healthcare providers lacked knowledge about PCS, which resulted in insufficient advice. For example, one patient underwent a 5-week intensive rehabilitation program that was later discovered to be too strenuous. Healthcare providers acknowledged their lack of knowledge to the patient, which undermined the patient’s confidence in the healthcare provider.

*“Then*,* after the second set of blood tests*,* when nothing came up again*,* the GP said*,* “I do not know; that is not very helpful*,* but I do not know what you have*,* so I’ll refer you to the nurse practitioner. Maybe talking to her will help.” So*,* I was essentially sent away again. At that point I thought: What do you know about COVID? There was already so much information available*,* but she just did not want to acknowledge it.”* – patient with poorer PEHS [2].

In one case, a patient was more knowledgeable than his healthcare provider and even updated his GP on treatment options that she was unaware of.

##### General practitioners

Although GPs acknowledge their lack of knowledge, they do not communicate this to their patients.

*“I think a lot of GPs*,* I heard that so often… and even specialists. Healthcare professionals all started with; ‘we do not know’ and that really does not help patients*,* in my opinion.”* – general practitioner [5].

According to the GPs, they face difficulty diagnosing PCS due to the absence of an accepted definition and rely primarily on patient-reported symptoms. One GP argued that PCS should be subdivided into somatically unexplained symptoms and somatically explained symptoms to get a better understanding of the disease. Explaining somatic symptoms may be possible in cases such as ICU patients, however, this was not always the case which can make diagnosis challenging.

#### Desirable aspects of healthcare delivery

##### Patients

Both groups stated that they were satisfied with the care from the occupational therapist and the physiotherapist. According to these patients, physiotherapists helped with relaxation and exercises to get them back on track in their daily lives, but also showed compassion, paid attention to how they were doing at home or at work, and emphasised that recovery comes in small steps.

Patients from both groups were less satisfied with the care provided by GPs. They mentioned that the main task of most GPs was to refer patients to other healthcare professionals, or only made a diagnosis. Patients from both groups also mentioned that GPs were busy at a later point during the COVID-19 pandemic, which resulted in limited consultation time and GPs did not contact patients proactively anymore. This gave patients the impression that GPs did not know what was going on with their health condition.

*“I have not heard from the GP for a year now. I have not been there either*,* but I would have liked it if she had made a phone call because she still gets reports from other healthcare providers. I am disappointed about that.”* – patient with poorer PEHS [2].

As a result, patients had the feeling that they had to be in control to get, for example, a referral to another healthcare provider.

Collaboration between healthcare professionals was perceived differently by patients. Some patients felt that when they were referred to another healthcare speciality, the care process had to recommence from the beginning, whereas others experienced that all involved healthcare providers were up-to-date about their status.

##### General practitioners

Maintaining long-term proactive contact with patients proved challenging for GPs. Initially, a GP proactively contacted patients due to the uncertainty surrounding COVID-19 as it was a novel disease at the time. As the pandemic continued, more patients experienced persistent symptoms (i.e. PCS) and proactive outreach was limited due to time constraints.

Some GPs agreed that they only referred people to other specialities and had no further supporting role. Other GPs indicated that they also conducted physical examinations, such as measuring saturation or listening to the lungs, in suspected PCS patients with presumed shortness of breath or extreme fatigue. These examinations are often requested by patients and often did not lead to a diagnosis. A GP mentioned that there should also be an emphasis on a healthy lifestyle and mental relaxation in the care process.

*“With some patients*, *I am already taking a more proactive approach […]*,* in the sense of emphasizing a healthy lifestyle*,* mental relaxation*,* emptiness in the head*,* […] I am trying to connect with the patients whom I fear are going out of balance due to a COVID-19 infection.”* – general practitioner [5].

A mental health nurse practitioner (MHNP) can help with this by calling vulnerable patients preventively.

According to the GPs, people with good PEHS require support in distributing their energy by planning daily activities, whereas the group with poorer PEHS require support in activation and a focus on care and support for their pre-existing conditions. Most GPs mentioned that the GP can take control of the care process for PCS patients because people trust their GP and they come to the doctor with their first symptoms. Some other GPs question if GPs are suitable for managing patients with unexplained somatic symptoms due to their high workload. Despite patient needs, lengthy conversations may not be possible. Thus, a GP stressed the need for longer consultation times for PCS patients.

*“At one point in practice*,* I did mention for post-COVID syndrome related questions*,* I need at least half an hour*,* because sometimes they would slip in a five-minute phone call in between. Well*,* that’s not feasible. And if I was lucky*,* I’d have twenty minutes*,* which could then stretch to half an hour. That way*,* you can make some progress*,* at least.” –* general practitioner [4].

The GPs also mentioned that they sometimes collaborated with other healthcare providers for the care of PCS patients, for example with the physiotherapist for a referral of the patient to secondary care. When the patient’s condition deteriorated, the physiotherapist contacted the GP for a consultation about the patient.

#### Discordance between patients’ experience and advice from healthcare providers

##### Patients

Most patients with poorer PEHS experienced a discordance between care advice received from healthcare providers and their own experiences. Healthcare providers advised taking rest, while patients preferred a more active approach, resulting in not feeling recovered and understood. Another patient experienced that increasing the physical intensity during physiotherapy did not help her to recover and only resulted in more fatigue. However, some caregivers provided sufficient advice, such as not crossing limits and building up physical condition slowly.

Some patients mentioned that there was insufficient recognition from healthcare providers, for example, GPs thought that the symptoms were related to another disorder, or GPs attribute the symptoms to burnout. Some patients received understanding after a while and had the feeling that they were taken seriously. However, others immediately felt acknowledged.

*“The GP took my symptoms seriously. He also asked me how it went*,* what I did about it*,* and what I could do about it. He just started the conversation seriously*,* which made me feel recognized.” –* patient with poorer PEHS [3].

##### General practitioners

The importance of taking PCS patients seriously and acknowledging their symptoms was emphasized by GPs.

*“If the symptoms fit with PCS*,* then mention that more people have PCS. That it is a well-known phenomenon*,* but that we do not yet know how it ends with everyone.”* – general practitioner [6].

Some GPs who doubted the existence of PCS never conveyed this scepticism to patients and mentioned that even though symptoms are elusive, naming the disease gave patients guidance on the symptoms. And, thus, the task of GPs was to help people even though there is still a lot unknown about PCS.

## Discussion

This interview study examined the experiences of Dutch post-COVID syndrome (PCS) patients and general practitioners (GP) regarding the aspects of living with PCS and the care for PCS patients. In this study, we distinguished between people with poorer PEHS and good PEHS. These types of patients varied in terms of: (1) aspects of living with PCS, as patients with good PEHS mainly experienced symptoms during overstimulation, while those with poorer PEHS generally experienced exhaustion constantly, and (2) healthcare experiences, as GPs emphasized that both groups require different care support; people with good PEHS need help balancing their energy through planning daily activities, whereas the group with poorer PEHS needs stimulation to an active lifestyle and focus on pre-existing problems. Although GPs emphasized the importance of taking patients seriously and acknowledging their symptoms, some patients indicated that GPs doubt the existence of PCS, resulting in insufficient recognition. In addition, GPs felt that they did not have a complete image of which of their patients had PCS because not all patients consulted them. Finally, our study revealed a discrepancy between the experiences of GPs and patients regarding the care provided to PCS patients, including ineffective care advice and the role of the GP in the care process.

### Comparison with literature

A scoping review regarding the management of PCS in general practice showed similar results as our study [33]. An example is the uncertainty of GPs to diagnose patients with PCS because many different PCS definitions have emerged and there is an overlap with other health conditions [34]. In literature, it was emphasized that it is important for GPs to listen to patients and show understanding and empathy [14, 35, 36]. Initial assessments of physical and/or psychological functioning can be guided by GPs and can play a key role in ruling out alternative diagnoses and serious complications [36]. Moreover, GPs can help patients navigate the pathways of the healthcare system [36, 37]. However, a discrepancy with the systematic review is that our study shows that patients experienced limited access to care facilities [36, 37]. Therefore, GPs in our study may not have been cognizant of their potential care role for these patients. Our addition to the existing literature is that we distinguished between people with poorer PEHS and good PEHS, as these patients experience symptoms and care needs differently. This requires a different approach for healthcare providers; people with good PEHS benefit from taking rest and balancing their energy instead of being activated to do more. The study of Krishna et al. shows through an integrated network biology approach the relationship between COVID-19 and pre-existing diseases such as cancer, neurological disorders, cardiac disorders, pulmonary diseases and hypertensive diseases, with regard to multiple organ damage [38]. Therefore, the awareness of the existence of two types of patients (PCS patients with poorer PEHS and PCS patients with good PEHS) and their different care needs could contribute to more appropriate care and for patients to feel acknowledged.

Patients in our study emphasized that they felt stigmatized by caregivers and by people who were not close to them, which was in line with a previous study [39]. Several studies focusing on other infectious diseases, such as Ebola, tuberculosis, and HIV, have shown that patients who fear stigma may experience difficulties in accessing care and treatment, leading potentially to poorer health outcomes [40–42]. The stigma caused by healthcare professionals diminishes the feeling of empowerment among patients and can cause patients to avoid care and treatment [43, 44]. Effective doctor-patient communication is crucial, where the focus should not be on attributing the disease burden, but on the acknowledgement of PCS by GPs, good listening, confirming patients’ suffering and ensuring continuity of care [44, 45]. A person-centred approach can help improve collaboration and co-production between GPs and PCS patients for better health outcomes [46] and thus plays an important role in coping with or recovering from PCS.

In our study, GPs had the feeling that PCS patients who actively reach out to their GP are likely the only ones known to them, leading to unequal access to care for those who do not seek help. However, these patients could benefit the most from support. Patients who avoid care are often characterized by low health self-efficacy and are less experienced in both receiving quality care and getting assistance for health-related uncertainties [44]. In our study, GPs mentioned that it is important to encourage PCS patients to remain positive by offering the perspective that the largest group of patients recover. However, patients who avoid care with their GPs will never receive this advice. Therefore, it is difficult to organize the care they need for everyone, and patients who avoid care require a specific approach. For example, GPs or nurse practitioners need to gain insight into which patients are avoiding care by proactively reaching out to vulnerable patients. They should carefully emphasize these patients when it is critical to seek care or GPs should adjust the care plan for those patients [47]. Support groups, i.e. an organization that supports people with long-term COVID symptoms (C-support), can emphasize that PCS patients can also consult their GP for help and, therefore, better coordination is needed between support groups such as C-support and GPs. Finally, decision support tools and telephone or web-based resources can improve PCS patients’ self-reliance and self-management of PCS and, thereby, help avoid unnecessary care [47].

There was a discrepancy between the experiences of PCS patients and GPs regarding care. Patients with PCS were dissatisfied with GP care and felt referred to other providers only, while GPs argued they provided physical exams and mental health conversations. Wrong advice caused confusion, such as rest when action was needed or too much physical intensity. These discrepancies may be due to the variety of PCS patients with different symptoms and care needs. A study towards the recognition of patients with medically unexplained physical symptoms (MUPS) by general practitioners showed also varying care and support needs in different types of patients (e.g. anxious, unhappy, passive, distressed or puzzling patients) [48]. Many symptoms of PCS are unexplained, making it similar to MUPS. Therefore, GPs need to recognize the types of PCS patients and their care needs.

### Strengths and limitations

This study had some notable strengths. To the best of our knowledge, this is the first study in the Netherlands investigating patients’ and GPs’ perspectives on aspects of living with PCS and healthcare needs and experiences. This created different perspectives, resulting in richness of the data. The topic lists in our study were reviewed by two experts in qualitative research, who were diagnosed with PCS themselves. This ensured that the topic list was properly tailored to the needs of PCS patients. Our study is part of a larger COVID-19-mixed method study. The Nivel Corona Cohort [21] and a cohort extracted from the Nivel Primary Care Database (Nivel-PCD) [24] provided insights that support this study; such as the variety of complaints in PCS patients.

This study has some limitations as well. While we utilized purposive sampling to select a diverse range of interviewees for data saturation, there is still a possibility of missing some perspectives. The care process for PCS patients involves several care providers, each with their unique insights and experiences, and thus, future research should explore the perspectives of other healthcare providers besides GPs. Despite using purposive sampling, we did not reach PCS patients with a migration background and not all PCS patients could participate in the interviews, because they were unable to participate in the interview as a result of their PCS symptoms. As a result, we might lack insights into the experiences of these patients, for example, with access to healthcare.

### Implications for practice and research

The study’s findings provide insight into how care for patients with PCS is delivered and what the experiences are from both the patient’s and the general practitioner’s perspectives. Acknowledgement of PCS by GPs is important for patients and plays an important role in coping with or recovering from PCS. In addition, GPs should be aware of the differences between two or more types of patients and their care needs. A multidisciplinary, person-centred approach is important, irrespective of the group to which a patient belongs, due to the variety of symptoms and emotional aspects of PCS. To ensure vulnerable patients with poor health status receive the necessary care, GPs and nurse practitioners should proactively reach out to those who are avoiding care. Coordination of the care process by a trusted GP can benefit all types of patients, together with evidence-based protocols to guide patients to appropriate care pathways. Therefore, there is a need for evidence-based protocols for different healthcare providers to navigate patients to the right care pathways. This approach can be supported by using GP guidelines as a basis [26].

Monitoring the views and perspectives of both GPs and patients is crucial, given the ever-changing care landscape. Future research should also include interviews with other healthcare professionals involved in PCS patient care, such as nurse practitioners, physical therapists, occupational therapists, pulmonologists, and rehabilitation therapists. Finally, quantitative research examining the care pathways of PCS patients can complement our findings, enabling the establishment of optimal care pathways for a large group of PCS patients.

## Conclusions

This study showed that two groups of PCS patients can be distinguished; individuals with good PEHS and individuals with poorer PEHS. Awareness of the existence of these types or more types of PCS patients and their different care needs by GPs could contribute to more appropriate care and for patients to feel acknowledged in coping with or recovering from PCS. These differences make a multidisciplinary person-centred approach important. Moreover, GPs or nurse practitioners should proactively reach out to vulnerable patients with poor health status who avoid care to ensure they obtain the care they need. The coordination of the care process can be done by a trusted GP. The findings presented here can provide a first insight into practical guidelines for providing care to patients with PCS.

## Electronic supplementary material

Below is the link to the electronic supplementary material.

Supplementary Material 1

Supplementary Material 2

## Data Availability

The raw data underlying this article will not be shared due to the anonymity of the participants. Reasonable requests for de-identified raw data will be considered by the corresponding author.

## Declarations

### Authors’ information

CR is a PhD student at Nivel, Netherlands Institute for Health Services Research and PhD student at Tranzo, Tilburg University, the Netherlands.

BK is a general practitioner and the leader of the research program General Practice Care at the Nivel, Netherlands Institute for Health Services Research.

RvdH was a junior researcher at Nivel, Netherlands Institute for Health Services Research and is currently a PhD student at the University Medical Center Groningen, the Netherlands.

LB was a post-doc researcher (PhD) at Nivel, Netherlands Institute for Health Services Research and is currently an Information analyst at Dutch Hospital Data, the Netherlands.

LvD is the coordinator of the research program Pharmaceutical Care at Nivel, Netherlands Institute for Health Services Research and honorary professor ‘Pharmacy health services research’, at the University of Groningen, the Netherlands.

RV is the coordinator of the research program Learning Health System at Nivel, Netherlands Institute for Health Services Research and endowed professor ‘Transparency in healthcare from a patient perspective’ at Tranzo, Tilburg University, the Netherlands.

IB is a senior researcher (PhD) at Nivel, Netherlands Institute for Health Services Research, the Netherlands.

## Abbreviations

COREQ: Consolidated criteria for Reporting Qualitative research
GPs: General practitioners
MHNP: Mental health nurse practitioner
MUPS: Medically unexplained physical symptoms
NHG: The Dutch College of General Practitioners
Nivel-PCD: Nivel Primary Care Database
PCS: Post-COVID syndrome
PEHS: Pre-existing health status (PEHS)
SBSQ: Set of Brief Screening Questions
SES: Socioeconomic status
