# Supplementary material for: Care needs of patients with the post-COVID syndrome in Dutch general practice: an interview study among patients and general practitioners
Source: BMC Prim Care. 2024 Sep 28;25:355. doi: 10.1186/s12875-024-02597-w (PMC11439229; doi:10.1186/s12875-024-02597-w)
Supplement: Supplementary file 1 — Supplementary Material 1 [file 12875_2024_2597_MOESM1_ESM.pdf]

Additional file 1

Topic list patient interviews

1. General information

- Age
- Residence
- Urbanisation (city/village/rural)
- Family situation
- Work situation
- General health (other conditions/chronic diseases)
- Migration background

2. Own definition of Long COVID / post-COVID syndrome

For this study, we interview people with long-term COVID-19 symptoms. That is called Long COVID or the post-COVID syndrome, but we do not know exactly what that means.

- Are you familiar with the term Long COVID/post-COVID syndrome?
- Do you have Long COVID/post-COVID syndrome? How did you come to that conclusion? Do you think the diagnosis is accurate? Do you ever doubt it? What symptoms or difficulties are you experiencing? How severe are they? How long have they been lasting?

3. COVID-19 acute phase (referring to the period when you first contracted COVID-19)

- When did you become infected with the COVID-19 virus?

- How did you find out you had COVID-19? Were you tested? If so, what kind of test and what variant of COVID-19 did you have?

- What changed in your situation after you were diagnosed with COVID-19? How did you feel about it?
  - o Health (COVID-19 symptoms, other conditions)
  - o Emotions/mental health (anxiety, gloom, insecure, lonely)
  - o Contact with healthcare providers (which? how (remotely)?)
  - o Contact with relatives
  - o Caring for loved ones and others (fear of contamination)

- Did you receive any treatment at the time? If so: where, by whom, and how? Was hospitalization necessary? Did you do things yourself to get rid of the symptoms?

- Was a follow-up program discussed during or after the treatment?
  - o Were any follow-up appointments scheduled? If so, with whom and in what form (remotely)?
  - o Did you know in which cases you had to contact them and with whom?

- Do you remember what information you received and how it was presented?
  - o What did you think of the way the information was conveyed?
  - o How clear was the information?
  - o Did you understand all the information well?
  - o Did you miss certain information?

- Did you know at the time that you could get long-term COVID-19 symptoms? If so: how did you know and how did you feel about it? If not, would you rather have known, how would you have known?

- Have you been infected with COVID-19 once or multiple times?

4. COVID-19 chronic phase (longer than three months after first symptoms)

- Do you still experience symptoms of COVID-19 at the moment? If not, how long did it last for you?

Symptoms

- Somatic

o Which symptoms do you experience exactly?
o Nature, extent, seriousness (if applicable: tractus history)
o Specific symptoms (NHG Standard 2022)

 Sleep problems
 Indications of muscle loss: unintentional weight loss, muscle weakness, insufficient balance, reduced nutrient intake (due to lack of appetite, loss of smell and/or taste or gastrointestinal problems)

o Duration (still experiencing symptoms at this moment?)
o Continuous or intermittent?
o Medication use
o Change in symptoms after vaccination/booster/second infection?
o Comorbidity, chronic diseases, general health

- Cognition

o Do you have an idea why your symptoms persist for so long?
Problems before COVID-19 (life events, illness, work)
o Do you think you can do something yourself to alleviate your symptoms? What exactly?
If so, have you tried that?
o Do you think that others can do something to alleviate your symptoms?

- Emotions

o How do the symptoms affect you emotionally?
anxious, depressed, hopeless, worried
o What exactly makes you feel that way?
long duration, hopelessness, lack of understanding/support

- Behaviour

o What do you do if your symptoms are very severe?
o Are there things you avoid because of your symptoms?
o Do you do anything to reduce your symptoms?
o Tips for other people with long-term COVID-19 symptoms: what is good to do and what not?

- Social

o Do you have to adjust your life due to your symptoms? (work, hobbies, private life)
o How do people in your environment react to your symptoms?

o Do the symptoms affect your social life?
o Impact on work
o Consequences for financial situation
o Contact with other people who experience long-term COVID-19 symptoms?

<u>Support (formal and informal)</u>

- From whom did you receive professional care for your long-term COVID-19 symptoms?
General practitioner (GP), home care, hospital, other (e.g. homeopathy, acupuncture)

- How did you experience this care for your long-term COVID-19 symptoms?
o What went well and what did not go well?
o Healthcare providers who have helped well: what did they do best?
o Healthcare providers who did not help well: what did they not do for you?
o How did you experience the collaboration between healthcare providers?
o Was there a coordinating care provider?

- Did you receive other support, for example from family or friends? If so, how did you experience this support?

- Did/would you (also) like to receive other help or support?
What kind of help? From who?

- What could healthcare providers do to better help you?

- Did/do you feel heard by healthcare providers?

- What could others do to better help you?

- What could society do to better help you?

- Are there any questions that bother you but that you have not received answers to?
If yes which one?

<u>Information</u>

- Have you received any information about your long-term COVID-19 symptoms?

- If so, what and from whom? Did it help you?
o How do you feel about the way it was provided?
o How clear do you find the information?
o Can you understand all the information well?
o Are you missing certain information?

5. Timeline

Summarize and discuss chronology, identify specific time points; contamination, symptoms (onset and changes), important events

6. Closing Questions

- Do you think you still have COVID-19 at the moment?

- When could you say that you have recovered?
symptoms, functioning, social life, work

- What is the biggest difference when comparing your health and life to the period before you got COVID-19?

Topic list general practitioners interviews

1. General information

- Age
- Work experience
- Urbanisation of work (city/village/rural)
- Type of practice (solo/duo/group)
- Patient population (vulnerable groups, elderly, limited health literacy, low SES, migration background)

2. Experience with long COVID (post-COVID syndrome)

- When did you become familiar with Long COVID (post-COVID syndrome, PCS)? How did you get information about it? And how come now?

- How many patients have you (had) approximately with Long COVID (PCS)?

- In your opinion, what causes these patients to have from Long COVID (PCS)? (and not others) *Symptoms or impairments? Severity? Duration?*

- Do you ever doubt whether these patients have (had) Long COVID (PCS) or is it completely clear?

- What medical actions did you perform on these patients (diagnosis, treatment, referral)?

- How did you support these patients? *Do you distinguish between vulnerable groups/patients?*

- When do you consider a patient to have recovered from Long COVID (PCS)? Criteria? *symptoms, functioning, social, work*

- How would you define Long COVID (PCS)? *There is no clear definition yet.*

3. Communication

- Do you discuss the possibility of developing Long COVID (PCS) with acute COVID patients you see? *Do you discuss this with the patient at that time? If so, what do you tell them; if not, why not? Do you make a distinction between patients? If so, how?*

- Do you talk to patients about Long COVID (PCS) during or after the acute phase? At which moment? What do you tell them?

- To what extent do you keep in touch with patients who have had acute COVID-19? How do you do that exactly? Initiative from the GP or the patient?

- Do you feel that all patients with possible Long COVID (PCS) will contact you themselves? If not, how do you notice that? Any ideas for improvement?

4. Risk groups

- Do you think that certain types of patients have a higher chance of getting Long COVID (PCS)? Or suffer more? Which groups are they?
  - o Comorbidity
  - o Limited social network
  - o Communication problems

o Vulnerable groups in grant application: children, the elderly, pregnant women, people with limited health literacy, low socioeconomic status, migration background and/or chronic conditions.

- Do you think these patients need more attention than other patients with COVID-19? If so, at what times (in the acute phase or after? or before?) Why? Why not?

- In your experience, are there patients who are less likely to seek help with acute COVID-19? If so, which patients are they? And to what extent are the patients who have are at increased risk of Long COVID? Why is that?

5. Collaboration with other disciplines

- How is your collaboration with other disciplines when it comes to patients with COVID-19? What does that collaboration look like?
Both acute COVID-19 and Long COVID (PCS)
Both primary and secondary care
Social domain

- Do you think that Long COVID should be treated primarily by the GP?
When and when not?

6. Bottlenecks and solutions

- What are the most important bottlenecks and solutions for you regarding vulnerable patients with Long COVID (PCS) concerning:
o Good care?
o Good information provision and communication?
o Collaboration with other disciplines?

7. Development over time

- Looking back at earlier stages of the COVID-19 pandemic, are there things you are doing or would do differently now regarding patients who have or may develop Long COVID (PCS)?

8. Closing Questions

- Research question: What barriers do you experience in the follow-up and care for patients with long-term COVID-19 symptoms, in particular patients from vulnerable groups? In other words, what are the challenges you face in caring for these individuals?

- What advice do you have for other GPs regarding the care for patients with long-term COVID-19 symptoms?
